# NF90 stabilizes cyclin E1 mRNA through phosphorylation of NF90-Ser382 by CDK2

**DOI:** 10.1038/s41420-020-0236-9

**Published:** 2020-01-22

**Authors:** Donglin Ding, Huixing Huang, Quanfu Li, Wenbo Yu, Chenji Wang, Haijie Ma, Jiaxue Wu, Yongjun Dang, Long Yu, Wei Jiang

**Affiliations:** 1 Key Laboratory of Metabolism and Molecular Medicine, the Ministry of Education, Department of Biochemistry and Molecular Biology, School of Basic Medical Sciences, Fudan University, Shanghai, China; 2 State Key Laboratory of Genetic Engineering, School of Life Science, Fudan University, Shanghai, China; 3 Department of Biochemistry and Molecular Biology, Mayo Clinic College of Medicine, Rochester, MN USA; 4 Laboratory of Cytobiology and Molecular Biology, The Affiliated Zhoushan Hospital of Wenzhou Medical University, Zhoushan, Zhejiang China

**Keywords:** Diagnostic markers, Hepatocellular carcinoma, Prognostic markers, Tumour biomarkers

## Abstract

Nuclear factor 90 (NF90), an RNA-binding protein, has been implicated in regulating interleukin-2 (IL-2) and the immune response. It was recently reported that NF90 is upregulated in hepatocellular carcinoma (HCC) tissues and promotes HCC proliferation through upregulating cyclin E1 at the posttranscription level. However, the regulation of NF90 in HCC remains unclear. We demonstrate here that cyclin-dependent kinase (CDK) 2 interacts with NF90 and phosphorylated it at serine382. Mechanistically, phosphorylation of NF90-Ser382 determines the nuclear export of NF90 and stabilization of cyclin E1 mRNA. We also demonstrate that the phosphorylation deficient mutant NF90-S382A inhibits cell growth and induces cell cycle arrest at the G1 phase in HCC cells. Moreover, an NF90-S382A xenograft tumor had a decreased size and weight compared with the wildtype NF90. The NF90-S382A xenograft contained a significantly lower level of the proliferation marker Ki-67. Additionally, in HCC patients, NF90-Ser382 phosphorylation was stronger in tumor than in non-tumor tissues. Clinically, phosphorylation of NF90-Ser382 is significantly associated with larger tumor sizes, higher AFP levels, and shorter overall survival rates. These results suggest NF90-Ser382 phosphorylation serves as a potential diagnosis and prognostic marker and a promising pharmacological target for HCC.

## Introduction

Hepatocellular carcinoma (HCC), the sixth most common malignancy, is one of the leading causes of death throughout the world^1,2^. Although surgical resection can provide great progress in HCC treatment, the recurrence and advancement of disease progression results in high lethality^3–5^. Hence, an understanding of the underlying mechanism of HCC pathogenesis can contribute to the development of effective strategies for HCC therapy.

NF90 is a well-known RNA-binding protein. In early studies, NF90 was found to play a key role in IL-2 regulation and the immune response. Studies have shown that NF90 binds to the promoter of IL-2 mRNA and thereby regulates IL-2 at the transcriptional level^6–8^. Further investigation indicated that NF90 can directly bind to the AU-rich 3′untranslated region (UTR) of IL-2 mRNA and stabilize the IL-2 mRNA and promote posttranscriptional upregulation^9,10^. In addition, the stabilization of other mRNAs containing the signature AU-rich region in 3′UTRs, including *p21*^*WAF1/CIP1*^ and *MyoD*, are also involved in the stringent regulation through NF90 binding^11^. Subsequently, NF90 has been extensively investigated in several biological processes, including transcription^12^, dsRNA/microRNA processing^13,14^, protein translation^15^, DNA repair, ribosomal subunits biogenesis^16^, host resistance to viral infections^17–19^, mitosis and cell cycle^20,21^.

Recently, its impacts on tumorigenesis and tumor progression are beginning to be revealed. NF90 has been reported as a novel tumor marker and its upregulation has been clarified in HCC^22–24^, urinary bladder cancer^25^ and cervical cancer^26^. Additionally, NF90/NF110 downregulation has been shown to be involved in ovarian carcinoma^27^.

Mechanistically, NF90 is involved in tumor progression mainly through affecting RNA processing. In HCC, our previous work showed that NF90 stabilized the cyclin E1 mRNA through direct binding. It upregulates cyclin E1 expression, accelerates cell proliferation, and promotes hepatocarcinogenesis^24^. Another study showed that NF90 regulates PARP1 mRNA stability in HCC cells^22^. In breast cancer, NF90 increases the level of polysome-associated vascular endothelial growth factor (VEGF) mRNA and promotes mRNA translation under hypoxic conditions^28^. The NF90-dependent promotion of angiogenesis has also been reported in human cervical cancer^26^ and colorectal cancer^29^ by regulating VEGF expression. The NF90/NF45 complex mediates E6 oncogene expression in human papilloma virus-transformed cervical carcinoma cells^30^.

miRNAs and long non-coding RNAs have recently emerged as key regulators in tumorigenesis, a process in which NF90 plays an important role. Overexpression of NF90-NF45 in HCC results in suppression of mature miR-7 and accelerates HCC cell proliferation^23^. NF90 is required for urinary bladder cancer cell stemness through controlling mi-145 biogenesis^25^. NF90 promotes colorectal cancer angiogenesis and metastasis via constituting a negative feedback loop with miRNA-590 to regulate VEGF^26^. NF90/NF110 facilitates DICER expression by controlling mi-1737 processing to act as a suppressor of ovarian carcinoma^27^. LincIN, a long non-coding RNA, mediates breast cancer cell invasion and metastasis through interacting with NF90^31^.

However, the precise mechanism driving NF90 to regulate RNA processing, such as tumor related mRNA cyclin E1 or VEGF, in tumor progression is still poorly understood. Various studies have suggested that NF90 phosphorylation is a key modification that is used to regulate its multiple functions^32–37^. Our previous work also supports the significance of NF90 phosphorylation, since both PKC and AKT can phosphorylate NF90-Ser647 to stabilize IL-2 mRNA^38,39^. However, NF90-Ser647 phosphorylation has no impact on cyclin E1 mRNA stabilization (data not shown), which indicates that there is another specific kinase involved in cyclin E1/cell cycle regulation by NF90. Notably, NF90 harboring the conserved phosphorylated site of cyclin-dependent kinases (CDKs) is regarded as a potential substrate of CDKs^33,37,40^. CDK2/cyclin E1 has the highest activity when the level of cyclin E1 reaches peak levels^41^, when cyclin E1 expression is under tight control.

Our current work demonstrates that NF90 is phosphorylated by CDK2/cyclin E1 at Ser382 and that CDK2 interacts with NF90. Sequentially, this phosphorylation contributes to the nuclear export of NF90 and stabilization of cyclin E1 mRNA. As a result, phosphorylation promotes cell cycle progression and HCC proliferation in vitro and in vivo. Our work reveals that NF90-Ser382 phosphorylation is positively associated with the survival of HCC pathogenesis and suggests a potential prognostic marker and target for HCC therapy.

## Results

### NF90 is a novel substrate of CDK2

**Fig. 1:**
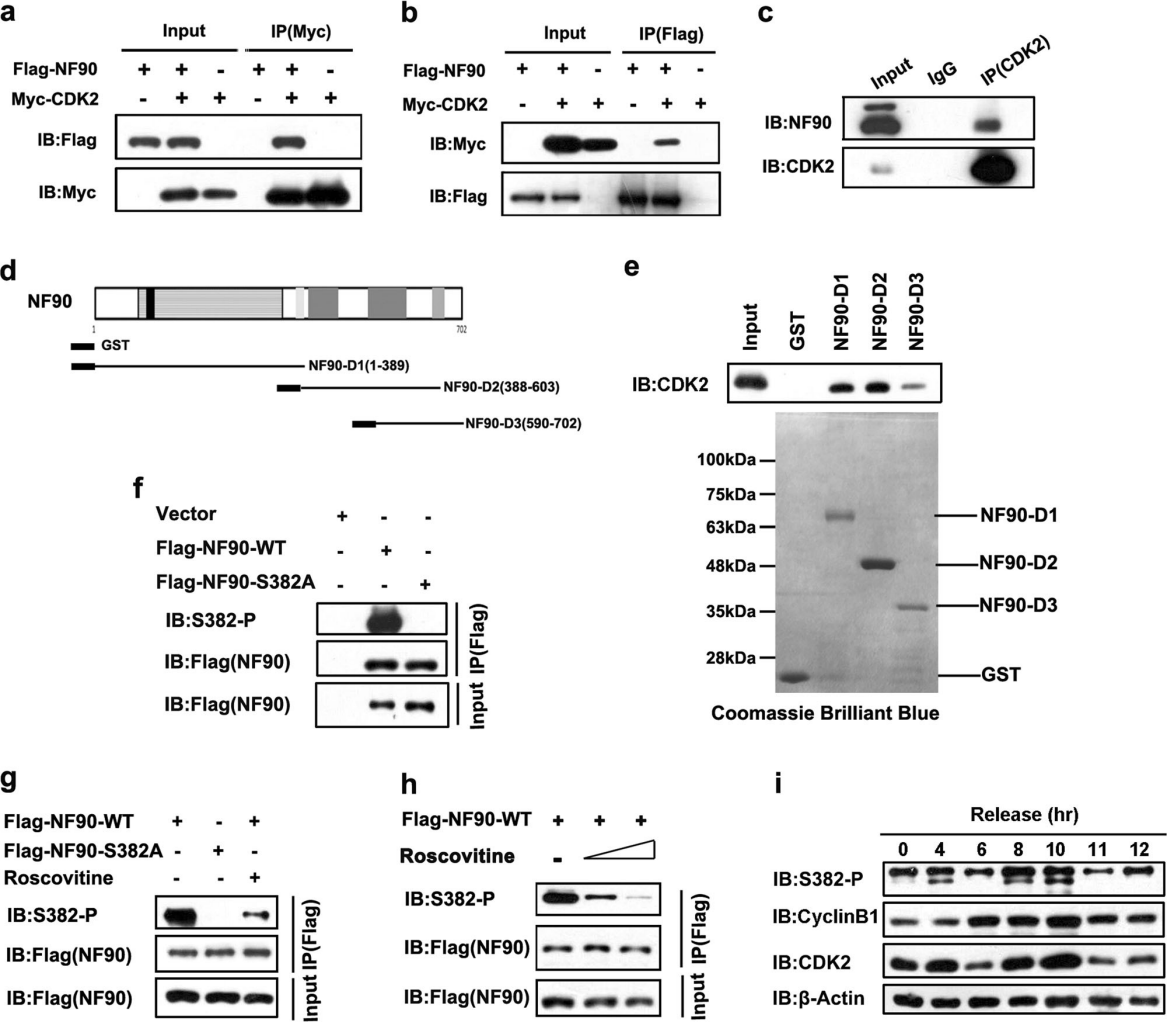
CDK2 interacts with and phosphorylates NF90. **a**, **b** Flag-NF90 immunoprecipitated with Myc-CDK2. After co-transfection with Flag-NF90 and Myc-CDK2 plasmids, HEK293T cell lysates were immunoprecipitated by either Flag or Myc antibody then subjected to western blot (WB) analysis. **c** Association between endogenous NF90 and CDK2. Cell lysates were collected for immunoprecipitation with the CDK2 antibody and subjected to WB as shown. **d** Schematic view of truncates containing different domains of NF90. **e** GST-pulldown assay to show which domain of NF90 is involved in the interaction with CDK2. Truncates with GST tag were purified and incubated with cell lysate. **f** NF90-WT and NF90-S382A plasmids were transfected into 293T cells. After 24 h, cells were lysed and NF90-WT and NF90-S382A were immunoprecipitated by M2 beads then subjected to WB. S382-P, antibody specifically recognizes the phosphorylated NF90-Ser382. **g** Phosphorylation of NF90-Ser382 was inhibited by roscovitine. 24 h after transfection, cells harboring NF90-WT were treated with 20 µM roscovitine for 24 h. IP and WB assays were performed as described above. **h** Cells were treated with 20 µM or 40 µM roscovitine for 24 h to detect the phosphorylation level of NF90-S382. IP and WB assays were performed as described above. **i** HeLa cells were arrested at the G1/S boundary twice by a double-thymidine block, then released and harvested at the indicated time points. Cell lysates were subjected to WB. β-actin served as a loading control.

To investigate the specific kinase and phosphorylation site regulating NF90’s function in cyclin E1 expression and tumor progression, we analyzed the predicted phosphorylation sites of NF90 (Fig. S1). Besides AKT and PKC, CDK had the potential to phosphorylate NF90. Considering that NF90 regulates cyclin E1 and promotes G1/S transition, we selected CDK2 to investigate. We performed immunoprecipitation (IP) assays to further determine whether NF90 is a target of CDK2. After co-transfection with Flag-NF90 and Myc-CDK2, HEK293T cell lysates were subjected to immunoprecipitation with Flag antibody or Myc antibody. Our results showed that Flag-NF90 immunoprecipitated with Myc-CDK2 (Fig. 1a, b). To explore whether endogenous NF90 and CDK2 interact with each other, cell lysates were collected to perform immunoprecipitation with the CDK2 antibody. As shown in Fig. 1c, NF90 could be detected in the CDK2 immunoprecipitates. In addition, Flag tagged NF90 pulled down endogenous CDK2 (Fig. S2a). These results clearly confirm that NF90 interacts with CDK2 in vivo. To determine the CDK2 binding region on NF90, we generated a set of truncated NF90 mutants (Fig. 1d) for GST pulldown assays. The result indicated that NF90 was directly bound to CDK2 and the binding region was mainly located in the N-terminal region of NF90 (Fig. 1e). In summary, we conclude that CDK2 interacts with the N-terminal region of NF90.

To find out whether NF90 is a new substrate of CDKs, we analyzed the protein sequence of NF90 and performed an alignment between human, rat and mouse species. NF90 sequences contain the consensus CDK-phosphorylation motif (KSPxK), and only one such motif exists (Fig. S2b). Given that CDK2/cyclin E1 complex is dynamically active in S phase entry and that NF90 stabilizes cyclin E1 mRNA to accelerate S phase entrance, we postulated that CDK2 phosphorylated NF90 to affect cell cycle and tumor progression. In vitro kinase assays and mass spectrometry (MS) identification were performed to determine whether NF90-Ser382 can be phosphorylated by CDK2/cyclin E1. GST-tagged NF90 was incubated with CDK2/cyclin E1 reaction buffer. After separating the complex by SDS-PAGE, NF90 proteins were subjected in-gel digestion with trypsin and detected by MS. The results showed that CDK2/cyclin E1 could phosphorylate NF90Ser382 in vitro (Fig. S2c).

**Fig. 2:**
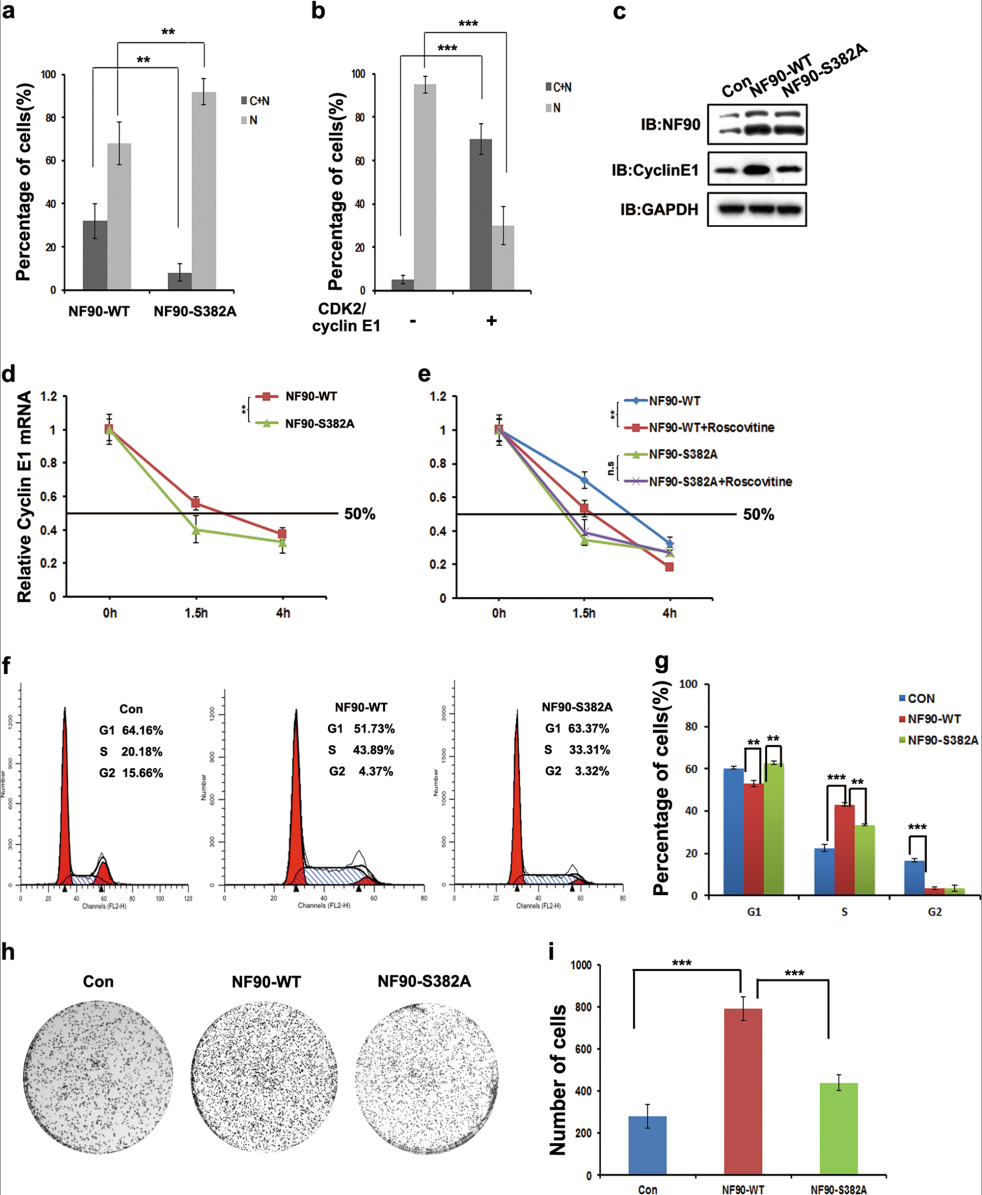
CDK2/cyclin E1 controls the nuclear export of NF90 to stabilize cyclin E1 mRNA and promotes cell proliferation in HCC cells. **a** NF90-WT and NF90-S382A display different nuclear export abilities. GFP tagged NF90-WT or NF90-S382A were transfected into HeLa cells. After 24 h, cells were fixed and stained with DAPI. C, cytoplasm; N, nuclear. Columns, mean (*n* = 30); bar, s.d. **p < 0.01. **b** Detection of nuclear export of GFP-NF90 after transfection with CDK2/cyclin E1 or not. C, cytoplasm; N, nuclear. Columns, mean (*n* = 30); bar, s.d. ***p < 0.001. **c** Huh 7 cells stably expressing NF90-S382A, NF90-WT or the control were constructed. Cyclin E1 expression in the stable lines was detected by WB. **d** The Huh 7 cells stably expressing NF90-S382A was used to examine the half-life of *cyclin E1* mRNA. Points, mean (*n* = 3); bar, s.d. **p < 0.01. **e** Increasing roscovitine significantly decreased *cyclin E1* mRNA half-time in Huh 7 cells stably expressing NF90-WT instead of NF90-S382A after 1.5 h incubation with actinomycin D. Points, mean (*n* = 3); bar, s.d. **p < 0.01. **f** Cell cycle analysis was carried out in Huh7 cells stably transfected with vector, NF90-WT, or NF90-S382A. The fraction of viable cells in the different cell cycle phases was analyzed by flow cytometry. Columns, mean (*n* = 3); bar, s.d. **p < 0.01; ***p < 0.001. **g** Colony formation assays were performed to assess the growth ability of Huh7 cells transfected with vector, NF90-WT, or NF90-S382A. Left, representative images of colony formation assay; right, analysis of the colony numbers. Columns, mean (*n* = 3); bar, s.d. ***p < 0.001.

In order to test whether NF90 was phosphorylated at Ser382 in vivo, a phosphorylation specific antibody (S382-P) was developed against a peptide containing phosphorylated Ser382. We first assessed NF90-Ser382 phosphorylation in 293T cells. Plasmids containing control and the phosphorylation deficient mutant NF90-S382A (serine to alanine mutation) were constructed as pFlag-NF90-WT and pFlag-NF90-S382A, respectively. Cell lysates were immunoprecipitated with Flag antibody and immunoblotted with S382-P and Flag antibody, respectively. The results indicated that S382-P antibody could specifically recognize the phosphorylated NF90 but not the phosphorylation deficient NF90-S382A mutant (Fig. 2f). Roscovitine, a potent inhibitor targeting CDKs, such as CDK2/cyclin E, CDK1/cyclin B and CDK2/cyclin A, underwent different preclinical and clinical studies^42^. As can be seen in Fig. 2g, NF90-Ser382 phosphorylation was obviously decreased after treatment with roscovitine. At the same time, NF90-Ser382 phosphorylation decreased with an increased dose of roscovitine (Fig. 2h). To further determine the NF90-Ser382 phosphorylation level during various stages of the cell cycle, the abundance of endogenous phosphorylated NF90-Ser382 was examined in HeLa cells at different time points throughout the cell cycle. HeLa cells arrested at the G1/S boundary were released to allow progression through the cell cycle synchronously. The NF90-Ser382 phosphorylation level was found to be consistent with the CDK2 protein level, which peaked at around 10 h after release (Fig. 2i). Overall, our data demonstrated that NF90-Ser382 is a target for phosphorylation and regulation by CDK2/cyclin E1.

### NF90-Ser382 phosphorylation promotes the nuclear export of NF90 to regulate the stability of cyclin E1 mRNA and promotes HCC cell proliferation

NF90 function in RNA processing is strictly dependent on its export from the nucleus. Our previous work showed that phosphorylation of NF90-Ser647 controls NF90 export to regulate IL-2 mRNA stability^38,39^. In this work, we found that Ser382 was located in the nuclear localization signal (NLS) of the NF90 protein (Fig. S2d). Hence, we hypothesized that NF90 nuclear export was dependent on NF90-Ser382 phosphorylation by CDK2/cyclin E1. We examined the distribution of phosphorylation of NF90-S382 in HeLa cells and found that, compared with NF90 WT, overexpression of phosphorylation deficient mutant S382A resulted in the significant decrease of NF90 cytoplasmic localization (Fig. S3a and Fig. 2a). Furthermore, in NF90-WT overexpression cells cotransfected with CDK2/cyclin E1, export of NF90 significantly increased (Fig. S3b and Fig. 2b). These data suggest that the CDK2-induced transfer of NF90 from the nucleus to the cytoplasm may be mediated by phosphorylation at Ser382.

As we previously demonstrated, NF90 plays an important role in cell proliferation by stabilizing cyclin E1 mRNA. In the present study, we investigated whether this effect was dependent on NF90-Ser382 phosphorylation. Stable overexpression of NF90-WT, NF90-S382A, and the control in the Huh-7 HCC cell line were constructed (Fig. 2c). The level of cyclin E1 protein was measured, and we found that the cyclin E1 protein level was significantly increased in NF90-WT overexpression cells compared to NF90-S382A and control cells. However, the NF90 phosphorylation deficient mutant didn’t show the same result. Next, stability of cyclin E1 mRNA level was compared between NF90-WT and NF90-S382A overexpression cells. As is shown in Fig. 2d, the cyclin E1 mRNA half-life in NF90-S382A was reduced in NF90-S382A compared with that in NF90-WT expressed HCC cells. Moreover, roscovitine significantly decreased the half-life of cyclin E1 mRNA in NF90-WT overexpressing cells. However, this effect was not observed in cells stably expressing NF90-S382A (Fig. 2e). These results indicate that inhibition of NF90-Ser382 phosphorylation attenuated the stability of cyclin E1 mRNA. We concluded that NF90-Ser382 phosphorylation plays an important role in stabilizing cyclin E1 mRNA.

**Fig. 3:**
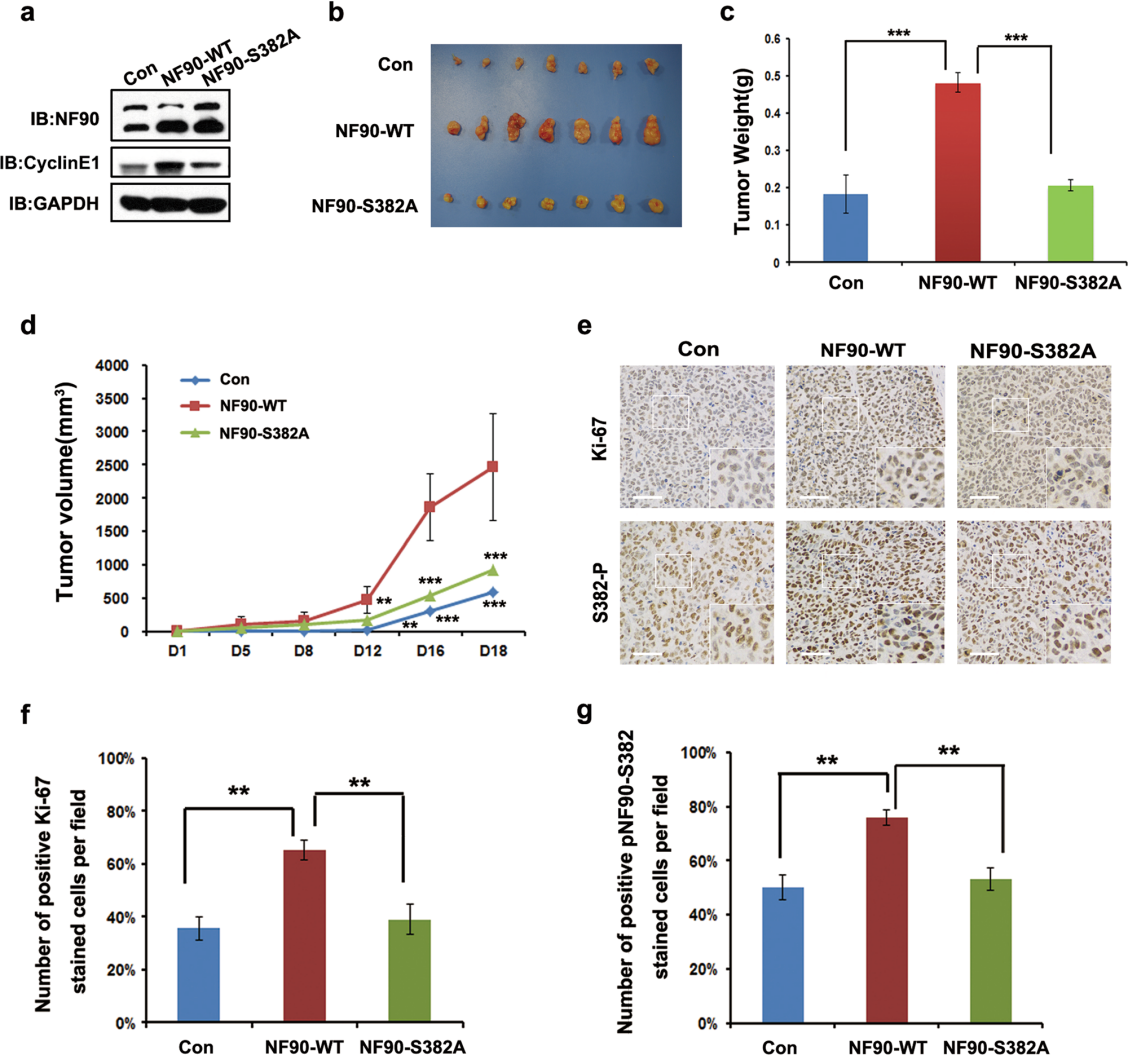
Deficiency of NF90-Ser382 phosphorylation inhibits tumor growth in vivo. **a** Cyclin E1 protein levels were detected in xenograft tissues. Three random xenograft tissues per group were selected and homogenized to examine cyclin E1 protein levels by WB. **b** Mice xenografts of Huh7 cells expressing NF90-WT, NF90-S382, or control. Tumor weight (**c**) and volume (**d**) were calculated among NF90-WT, NF90-S382 and control expressed Huh7 cells. **c** Columns, mean (*n* = 7); bar, s.d. ***p < 0.001 (**d**) Points, mean (*n* = 7); bar, s.d. **p < 0.01; ***p < 0.001. **e** Ki-67 expression was investigated by IHC analysis, scale bar, 25 μm. **f** Ki-67 positive cells were assessed in Huh7 cells expressing NF90-WT and NF90-S382. Columns, mean (*n* = 4); bar, s.d. **p < 0.01. **g** NF90-S382 phosphorylation positive cells were shown in Huh7 cells expressing NF90-WT and NF90-S382. Columns, mean (*n* = 4); bar, s.d. **p < 0.01.

To investigate the role of NF90-Ser382 phosphorylation in cell proliferation, we compared NF90-S382A with NF90-WT or control Huh-7 cells; overexpressing NF90-S382A exhibited the lowest growth ability (Fig. S3c), and a similar result was also seen in HEK293T cells (Fig. S3d and e). Cell cycle progression was then measured by flow cytometry, and we observed a significant increase in the S phase population for NF90-WT Huh-7 stable cells compared with the control (43.89 ± 3.63% vs. 20.18 ± 5.28%). However, NF90-S382A significantly impaired the effect, since the S phase population was 33.31 ± 2.13% (Fig. 3f, g). Furthermore, overexpression of NF90-WT obviously accelerated HCC cell colony formation compared with the control cells. However, NF90-S382A stably expressing cells inhibited colony formation compared with NF90-WT (Fig. 3h, i). Taken together, our data showed that the deficiency of NF90-Ser382 phosphorylation slows down the cell proliferation rate compared with wildtype in vitro.

### Deficiency of NF90-Ser382 phosphorylation inhibits tumor growth in vivo

To further assess the effect of NF90-Ser382 phosphorylation on cell proliferation in vivo, we carried out a nude mice xenograft assay. NF90-WT, NF90-S382A, and the control cell lines were subcutaneously injected into nude mice. NF90-WT/S382A protein expression in xenograft samples was first confirmed by WB. Cyclin E1 protein levels consistently increased in the NF90-WT xenograft tissue, but the NF90-S382A mutant cells did not show this effect (Fig. 3a). We found that the NF90-WT group promoted tumor growth in vivo, while NF90-S382A did not (Fig. 3b). In accordance with this observation, NF90-WT resulted in a significant increase in the weight and volume of tumors. However, the NF90-S382A mutation almost completely ameliorated this effect (Fig. 3c, d). Moreover, Ki-67 protein, a cell proliferation marker, was analyzed in tumor samples by immunohistochemistry (IHC) staining. We confirmed that Ki-67 expression in NF90-WT tumor samples was evidently increased, but NF90-S382A tumor samples exerted no visible difference compared with the control. Furthermore, Ki-67 expression was accompanied by NF90 S382 phosphorylation, as confirmed by IHC analysis (Fig. 3e–g). These results suggest that HCC cell proliferation in vivo requires the phosphorylation of NF90-Ser382.

### NF90-Ser382 phosphorylation is negatively correlated with the survival of HCC patients

**Fig. 4:**
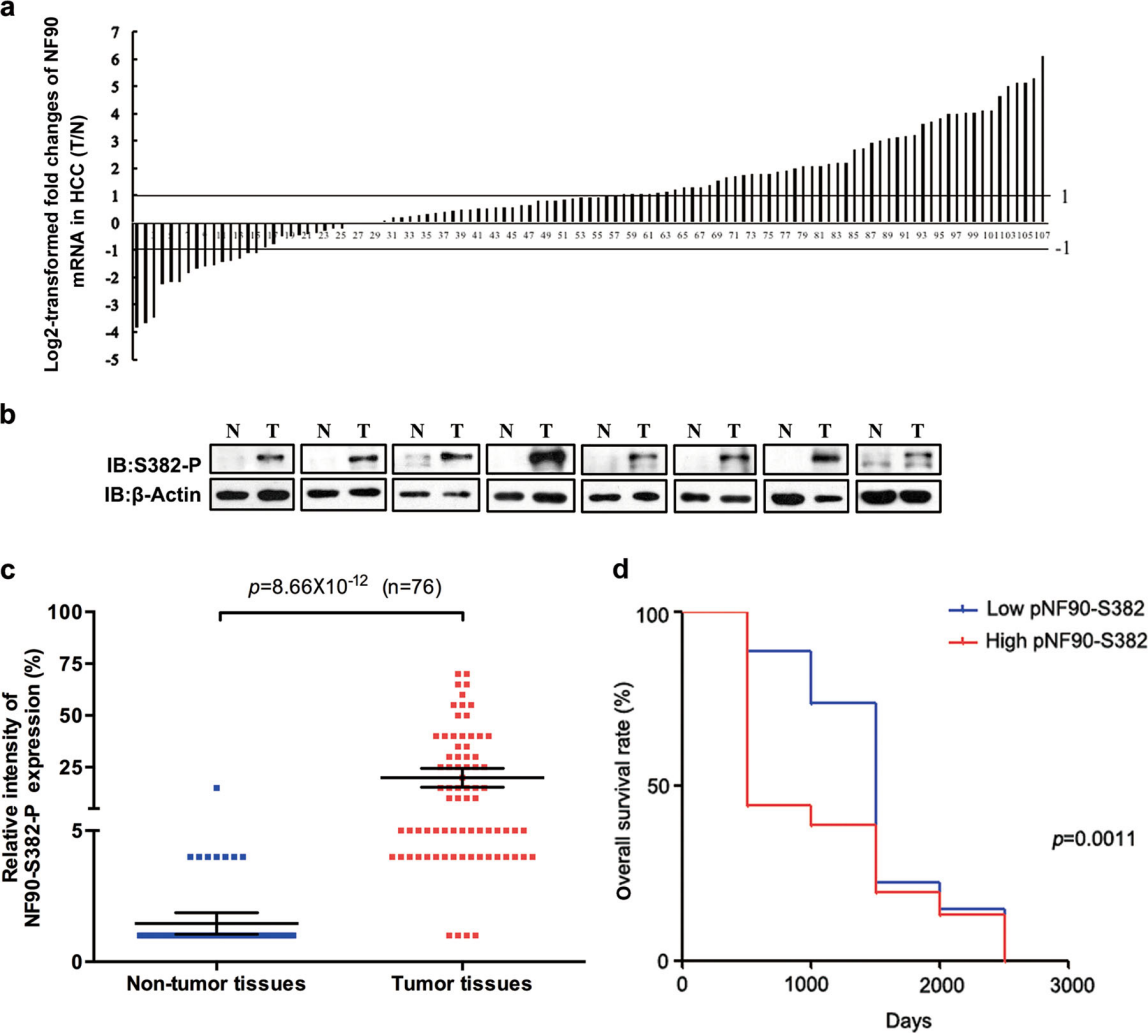
High expression of NF90-Ser382 phosphorylation indicates poor prognosis of HCC. **a** NF90 mRNA expression levels were analyzed in 107 paired HCC specimens by qRT-PCR and normalized to *β2-microglobulin* (*β2 MG*). A two-fold change threshold was set for identifying significant changes in gene expression. T, tissue; N, non-tumor tissues. **b** The phosphorylation level of NF90-Ser382 in 8 paired HCC specimens. β-actin served as the loading control. T, tissue; N, non-tumor tissues. **c** Comparison of NF90-S382 phosphorylation levels in tumor tissues and non-tumor tissues. Intensity of the NF90-S382 phosphorylation level was calculated by ImageScope software. Scatter plots, mean (*n* = 76); bar, s.d. *p* value was shown in the picture. **d** Survival rate is correlated with NF90-S382 phosphorylation level. Low level of NF90-S382 phosphorylation was associated with longer survival.

The NF90 antibody recognized both NF90 and NF110, which was not specific for IHC analysis. To confirm the role of NF90 in human HCC, we surgically collected 107 pairs of HCC specimens with non-tumor adjacent normal tissues to perform qRT-PCR to evaluate the clinical relevance of NF90 expression. We found that NF90 was significantly upregulated more than 2-fold in 45.79% of the HCC specimens (Fig. 4a), a result that was similar with our previous findings^24^. High-level NF90 expression was associated with positive HBV e antigen expression in HCC tissues (Table 1). Considering that NF90-Ser382 phosphorylation promotes HCC progression in vitro and in vivo, we sought to further determine whether NF90-Ser382 phosphorylation was related to HCC clinical characteristics.

**Table 1:** Correlation between clinicopathological features of HCC patients and NF90 mRNA level.

Variation	Relative NF90 mRNA intensity in HCC (Tumor/Adjacent-non-tumor)		
	Low (T/N ≤ 0)	High (T/N > 0)	*p* value
*Gender*			0.951
Male	47	41	
Female	10	9	
*Age (years)*			0.962
≥50	10	33	
<50	5	17	
*HBV e antigen*			**0.014**
Positive	21	29	
Negative	26	12	
*Tumor size (diameter)*			0.277
≥5 cm	9	22	
<5 cm	6	28	
*Tumor number*			0.260
Single	9	37	
Multiple	3	5	
*Cirrhosis*			0.364
Yes	5	7	
No	44	35	
*AFP level (ug/L)*			0.837
≥20	24	26	
<20	28	28	
*Portal vein tumor thrombosis*			0.536
Yes	26	25	
No	23	17	
*Tumor encapsulation*			0.618
Yes	8	23	
No	7	27	
*Pathological differentiation*			0.754
I-II	11	35	
III-IV	3	12	
*TNM clinical stage*			
I	9	20	I-II 0.266
II	6	26	I-III 0.191
III	0	4	II-III 0.343

Chi-square test was calculated

Bold characters represent statistical significance (*p* < 0.05)

We investigated the expression of NF90-Ser382 phosphorylation in 8 pairs of HCC tumors with the corresponding adjacent normal tissues by WB. As shown in Fig. 4b, the NF90-Ser382 phosphorylation antibody was sensitive enough to detect NF90-Ser382 phosphorylation levels, which were evidently upregulated in tumor tissues compared with non-tumor tissues. We further analyzed NF90-Ser382 phosphorylation in 76 pairs of HCC tissues. We found that NF90-Ser382 phosphorylation was almost undetectable in non-tumor tissues, which was consistent with the WB results. In contrast, positive staining of NF90-Ser382 phosphorylation was detected in most HCC tissues (Fig. 4c). As shown in Table 2, high levels of expression of NF90-Ser382 phosphorylation were remarkably correlated with large tumor size (*p* < 0.0026) and high AFP levels (*p* < 0.0037). Additionally, we conducted an overall survival analysis to examine the correlation of the level of NF90-Ser382 phosphorylation with the survival rate. There were significant differences in the survival rates of all patients between the low- and high- NF90-Ser382 phosphorylation expression groups (*p* < 0.01), and high NF90-Ser382 phosphorylation was associated with shorter overall survival (Fig. 4d). These clinic pathological findings suggest that NF90-Ser382 phosphorylation is a key determinant promoting the progression of HCC.

**Table 2:** Correlation between clinicopathological features of HCC patients and NF90-S382 phosphorylation level.

Variation	Relative NF90-S382 phosphorylation intensity in HCC (Tumor/Adjacent-non-tumor)		
	Low (T/N < 2)	High (T/N ≥ 2)	*p* value
*Gender*			0.863
Male	23	41	
Female	4	8	
*Age (years)*			0.945
≥50	14	25	
<50	13	24	
*HBV e antigen*			0.733
Positive	20	38	
Negative	7	11	
*Anti-HCV*			0.190
Positive	27	46	
Negative	0	3	
*Tumor size (diameter)*			**0.003**
≥5cm	8	31	
<5 cm	19	16	
*Tumor number*			0.314
Single	20	41	
Multiple	7	8	
*Cirrhosis*			0.425
Yes	19	30	
No	8	19	
*AFP level (ug/L)*			**0.004**
≥20	15	42	
<20	12	7	
*Portal vein tumor thrombosis*			0.688
Yes	5	11	
No	22	38	
*Tumor encapsulation*			0.137
Yes	22	32	
No	5	17	
*Vascular invasion*			0.316
Yes	10	24	
No	17	25	
*Recurrence*			0.561
Yes	13	27	
No	14	22	
*Pathological differentiation*			0.068
I-II	18	17	
III-IV	9	32	
*TNM clinical stage*			0.181
I	6	7	
II	11	13	
III	10	29	

Chi-square test was calculated

Bold characters represent statistical significance (*p* < 0.05)

## Discussion

Loss of growth control is a hallmark of cancers including HCC. Although mammalian cells reveal highly conserved cellular processes in proliferation regulation, the molecular pathways controlling the steps throughout the cell cycle are usually abnormally activated in cancers. In the case of cancers, cyclins or CDKs often experience uncontrolled expression^43^. Major regulatory events leading to cell proliferation occur in the G1 phase, when CDK2 activity is induced at the G1/S boundary after binding to cyclin E1. Specific CDK/cyclin complexes regulate transition through the distinct phases of the cell cycle by phosphorylating phase-specific substrates. Retinoblastoma protein phosphorylation, which is performed by CDK2/cyclin E1, leads to the activation of the E2F transcription factor and cell cycle-promoted genes^44^. However, many of substrates of CDK/cyclin complexes remain to be clarified, especially the tumor cell proliferation-related proteins. Both CDK/cyclin complexes and their tumor specific substrates are thought to be good therapeutic target candidates.

In our previous study, we established a causal link between NF90 and elevated cyclin E1 mRNA in HCC cells and tissues^24^. In the current work, we further determined NF90 as a novel substrate of CDK2. CDK2/cyclin E1 phosphorylated NF90 at serine 382 (Fig. 1), which was the key step driving NF90–mediated stabilization of cyclin E1 mRNA and promoted HCC cell proliferation in vitro (Fig. 2) and in vivo (Fig. 3).

NF90 has been reported to be a substrate of AKT or PKC kinase in T cells. It was determined that NF90-Ser647 was phosphorylated by AKT or PKC upon CD3/CD28 or PMA stimulation, leading to NF90 export from nuclear and stabilizing IL-2 mRNA^38,39^. However, in HCC cells, NF90-Ser647 phosphorylation is not responsible for cyclin E1 mRNA stabilization and cell cycle progression. We sought to determine the specific kinase and corresponding phosphorylation site of NF90. NF90 has an NLS and usually locates in the nucleus. Export from the nucleus is the basis for NF90 regulating mRNA in cytoplasm. CDK2/cyclin E1 phosphorylates NF90 at serine 382, which happens to be in the NLS of NF90. It is reasonable that NF90-Ser382 phosphorylation results in NF90 export from the nucleus, which we showed by immunochemistry assay (Fig. S3a-b and Fig. 2a, b). In addition, the abundance of NF90-Ser382 phosphorylation is consistent with CDK2 protein levels throughout the cell cycle (Fig. 1i), suggesting that NF90 is under tight regulation by CDK2 during tumor cell proliferation.

We further examined the effect of NF90-Ser382 phosphorylation on cyclin E1 mRNA regulation and HCC progression. There was high expression of endogenous wildtype NF90 protein that could be converted to the phosphorylated form in HCC cells. In this case, the effect of the exogenous transfection of the NF90-Ser382 phosphorylated mutant will be offset. Based on this conundrum, we constructed the NF90-Ser382 phosphorylation deficient mutant in order to observe the effect. This mutation made cyclin E1 mRNA unstable (Fig. 2d) and it significantly decreased cell growth and arrested cells in the G1 phase compared with wildtype (Fig. 2f, g). Furthermore, the deficiency of NF90-Ser382 phosphorylation obviously slowed xenograft growth (Fig. 3). It is suggested that NF90-Ser382 phosphorylation contributes to HCC cells proliferation and progression.

Upregulation of cyclin E1 is found in a variety of tumors, including breast^45^, ovarian^46^, colorectal^47^ and HCC^48^. From chronic hepatitis to HCC, the multistep process involves frequent upregulation or amplification of cyclin E and many other cell cycle-related proteins^49^. In addition, sustained high-level expression of cyclin E1 can cause chromosome instability, which is conducive to tumor formation^50^. It is important to determine the molecular mechanism prompting cyclin E1 expression in such tumors.

We have found for the first time that NF90 is upregulated in HCC. Meanwhile, there is a significant positive correlation between NF90 and cyclin E1 protein levels in human HCC specimens^24^. Later studies have revealed increase of NF90 in several tumors. These studies indicate that NF90 can serve as a novel tumor marker and participate in tumorigenesis. We investigated the correlation between the clinicopathology of NF90 expression and HCC by qRT-PCR, which could precisely identify NF90 splicing isoforms excluding NF110. The results showed that HCC patients with high-level NF90 expression usually had a history of hepatitis B (Fig. 4a and Table 1). Given that NF90 has been highlighted for its involvement in host resistance to viral infections and that HCC develops on the basis of chronic hepatitis in a multistep process, the above result seems reasonable. To go deeper into the clinical correlation between HCC and the role of NF90 promoting tumor cell proliferation, we attempted to detect NF90-Ser382 phosphorylation levels in HCC tissues using a specific phosphorylation antibody. We found that NF90-Ser382 phosphorylation levels were upregulated in liver cancer, while phosphorylation at this site was nearly undetectable in non-tumor tissues (Fig. 4b, c). NF90-Ser382 phosphorylation was positively associated with tumor size and AFP level (Table 2). Moreover, high levels of NF90-Ser382 phosphorylation indicated poor overall survival for HCC patients (Fig. 4d). This result suggest NF90 phosphorylation by CDK2/cyclin E1 promotes HCC progression. It is speculated that the synergy between NF90 and CDK2 inhibitors might someday offer a new therapeutic strategy for the treatment of HCC.

In summary, our study revealed a feedback pathway in HCC that is dependent on NF90-Ser382 phosphorylation by CDK2/cyclin E1. NF90-Ser382 phosphorylation regulates the nucleus export of NF90 to stabilize cyclin E1 mRNA, accelerates the cell cycle progression, and promotes HCC cell proliferation. Overall, our work demonstrates that NF90 phosphorylation is a key determinant in NF90’s effect on HCC pathogenesis.

## Materials and methods

### RNA isolation and quantitative real-time PCR (qRT-PCR)

RNA was extracted from tissue samples or cells using Trizol reagent (Thermo Fisher Scientific, U.S.). The isolated RNA was then used to synthesize the complementary DNA by reverse transcriptase (Toyobo, Japan). The mRNA expression was assessed using the Lighter cycle 480II RT-PCR system (Roche, Switzerland) with SYBR Green Mix (Toyobo, Japan). Relative gene expression was normalized to the expression of internal control β2-microglobulin (β2-MG) before calculating gene expression levels.

### Plasmid construction, transfection and RNA interfering

NF90-S382A was constructed by introducing a site-mutation in the coding sequence of NF90-Ser382 with KOD-Plus-Mutagenesis Kit (Toyobo, Japan). The plasmids were transfected into cells by lipofectamine 2000 (Thermo Fisher Scientific, U.S.). Small interference RNA (siRNA) targeted to *NF90 mRNA* or negative control siRNA (NC siRNA) were cotransfected with INTERFER in transfection reagent (Polyplus, France).

### HCC tissue samples and tissue microarray assay (TMA)

HCC tissue samples with the corresponding adjacent non-tumor tissues were obtained from Qidong Liver Cancer Institute (Jiangsu province, China). Fresh specimens were immediately frozen and stored in liquid nitrogen for analysis after surgical resection from patients. Ethics permits were approved by the ethics committee of the local institute and informed consents were obtained from the patients involved in the study.

The commercially used TMAs containing a total of 80 pairs of HCC specimens with clinicopathological details were purchased from Zhuoli Biotechnology Co., Ltd (LVC1605, Shanghai, China). 76 pairs of HCC tissues were successfully stained. The clinicopathological characteristics of patients are available in Table 2. The relative intensity of positively expressed pNF90-S382 in both tumor tissues and non-tumor tissues was assessed by ImageScope software (Aperio Technologies, Vista, CA).

### Cell lines and cell culture

HEK293T, HeLa and Huh7 cells were grown in Dulbecco’s modified Eagle medium (DMEM) (Invitrogen, Carlsbad, U.S.) supplemented with 10% (V/V) fetal bovine serum (FBS) (Biowest, France) at 37 °C supplemented with 5% CO_2_ in an incubator. Mycoplasma in these cell lines were eradicated by related reagent (Plasmocin, Invivogen, U.S.) according to the manufacture’s recommendation prior to carrying out the experiments. All the cells were maintained and stored in Dr. Long Yu’s laboratory at Fudan University.

### In vitro kinase reaction and identification of phosphorylation sites by MS

A total of 2 μg of NF90-D1 peptide in reaction buffer (50 mM Tris-HCl, 150 mM NaCl, 0.05% Brij35 (Santa Cruz Bio., U.S.), 1 mM DTT, 10% glycerol (Sigma, U.S.), pH 7.5) was incubated with CDK2/cyclinE1 complex (Carna Biosciences, Japan) according to the manufacturer’s instruction. After incubation, the reaction buffer containing NF90-D1 peptide was denatured and loaded for protein electrophoresis. Once the indicated loading band progressed sufficiently far in the gel, the band was excised for MS identification, which was performed following the instruction of the Proteomics Technology Platform of State Key Laboratory of Genetic Engineering, Fudan University, Shanghai. Briefly, gel slices were digested at 37 °C for 8 h, followed by reduction in 10 mM DTT. Peptides were then isolated from gels and desalted with a MicroTrap C8 (Phenomenex, U.S.). The phosphopeptides were enriched for MS analysis as described^51^.

### Preparation of antibody targeted to phosphorylated NF90-Ser382

The antibody production was commercially customized by Youke Bio Ltd. Company (Shanghai, China). Briefly, the phosphorylated peptide C-DGEEK(pS)PSKK-NH_2_ and unphosphorylated peptide C-DGEEKSPSKK-NH_2_ were synthesized and used to immunize rabbits. The phosphorylated peptide and unphosphorylated peptide were coupled to the affinity column and purified using the antibody against phosphorylated NF90-S382 from the serum of immunized rabbits.

### Immunoprecipitation and western blotting

Cells were collected and lysed with 1 × NETN (0.5% NP-40, 20 mM Tris-HCl, pH 8.0, 10 mM NaCl, 1 mM EDTA) supplemented with proteinase inhibitor (cocktail, Roche). Cell lysates were collected by centrifugation and incubated with protein A/G beads (Sigma, U.S.) with the indicated antibodies for immunoprecipitation. Total proteins were obtained and separated by SDS-PAGE and transferred to a nitrocellulose membrane (GE health, U.S.). The antibodies used in immunoprecipitation or western blotting were as follows: anti-Flag (F3165, Sigma, U.S.), anti-Myc (M4439, Sigma, U.S.), anti-CDK2 (ab32147, Abcam, U.K.), anti-cyclin E1 (ab33911, Abcam, U.K.), anti-cyclin B1 (sc-245, Santa cruz, U.S.), anti-NF90 (ab225626, Abcam, U.K.), anti-GAPDH (AP50812, Abgent, U.S.), anti-β-Actin (sc-47778, Santa cruz, U.S.) and anti-pNF-90 (S382; Youke, Shanghai, China). Roscovitine was purchased from Sigma and dissolved in dimethyl sulfoxide (DMSO) (Sigma, U.S.) and then included in the growth inhibition assay.

Antibodies to phosphorylated proteins were diluted by 5% BSA (Sigma, U.S.) in TBST according to the manufacturer’s instruction.

### Synchronization

HeLa cells were cultured to 30% confluence and grown overnight. Thymidine (Sigma, U.S.) was added the following day into cells to a final concentration of 2 mM. Cells were incubated for 20 h, washed with 1 × PBS, and cultured in fresh DMEM plus 10% FBS. After a 9h-incubation, 2 mM thymidine was added to the cells for another 18h-incubation. Finally, cells were washed with 1 × PBS and released by adding fresh DMEM plus 10% FBS. Cells were collected at 0, 4, 6, 8, 10, 11 and 12 h for WB analysis starting at the beginning of release. CyclinB1 expression was used as a marker to assess cell cycle synchronization.

### Cell growth assay

Cell growth assays were carried out using CCK-8 reagent (Dojindo, Japan). In brief, the cells were placed at 1000 cells per well in 96-well plates and cultured in DMEM with 10% FBS. At specific times, optical density of cells was measured by a microtiter reader (Biotek, U.S.) at 450 nm after incubating with CCK-8 for 3 h.

### Flow cytometry analysis

Cells were trypsinized, washed twice with cold PBS, collected by centrifugation, and fixed with ice-cold 70% ethanol at −20 °C overnight. Cells were washed twice with cold PBS and centrifuged. Propidium iodide (BD Bioscience, U.S.) was added to the cell suspension in PBS and incubated for 15 min in the dark. After that, cells were subjected to cell cycle analysis by FACS Calibur flow cytometer (BD Bioscience, U.S.).

### Immunofluorescence and IHC

The transfected cells grown on the cover slides were washed with cold PBS, fixed with 4% paraformaldehyde, and permeabilized with 0.2% Triton-X. After four washing with PBST, cells were further stained by DAPI (Thermo Fisher Scientific, U.S.) and imaged with a Zeiss LSM710 microscope.

For IHC analysis, paraffin-embedded tissues were fixed on slides and then blocked with 5% donkey serum (Gibco, U.S.) for 45 min, followed by incubation with anti-pNF90-Ser382 antibody (Youke, Shanghai, China) or anti-Ki67 antibody (Abcam, U.K.) overnight at 4 °C. The horseradish peroxidase-conjugated secondary antibody was used and the Diaminobenzidine Substrate Kit (Sigma, U.S.) was applied for signal detection. All tissue sections were counterstained with hematoxylin (Sigma, U.S.).

### Subcutaneous xenograft study in nude mice

Xenograft assay were carried out using six-week-old female BALB/c nu/nu mice (Slac) in accordance with the Animal Care and Use Committee guidelines of Department of Laboratory Animal Science, Fudan University. Mouse was randomly subcutaneously injected with different Huh7 stable cell lines (control, NF90-WT and NF90-S382A, 4 × 10^6^ cells/mouse) and the developing tumor was measured every four days. The volume of tumor was calculated by the formula: volume = 0.5 × (long diameter) × (short diameter)^2^. Tumors were excised after thirty days post injection. After that, tumors were fixed with 4% paraformaldehyde and subjected to immunohistochemical analysis.

### Statistical analysis

All assessments were carried out using GraphPad Prism software (5.0, San Diego, CA). Analyses of phosphorylated NF90-Ser382 expression in patients with clinicopathological details were calculated by Pearson’s chi-square test. Survival rate was determined with the log-rank (Mantel-Cox) test. Results of functional assays are shown as mean ± standard deviation (s.d.) from three or more independent experiments, and differences between two groups were evaluated with two-sided Student’s *t* test; *p* < 0.05 was considered statistically significant.

## Supplementary information

Supplementary Material

Detailed Attribution of Authorship

Supplementary Material

Supplementary Material

Supplementary Material
