# Supplementary material for: NF90 stabilizes cyclin E1 mRNA through phosphorylation of NF90-Ser382 by CDK2
Source: Cell Death Discov. 2020 Jan 22;6:3. doi: 10.1038/s41420-020-0236-9 (PMC7026180; doi:10.1038/s41420-020-0236-9)
Supplement: Supplementary file 1 — Supplementary Material [file 41420_2020_236_MOESM1_ESM.docx]

**NF90 stabilizes cyclin E1 mRNA through phosphorylation of NF90-Ser382 by CDK2**

Donglin Ding^1,2,3*^, Huixing Huang^1,2*^, Quanfu Li^1*^, Wenbo Yu^2^, Chenji Wang^2^, Haijie Ma^4^, Jiaxue Wu^2^, Yongjun Dang^1^, Long Yu^2^ and Wei Jiang^1^

^1^Key Laboratory of Metabolism and Molecular Medicine, the Ministry of Education, Department of Biochemistry and Molecular Biology, School of Basic Medical Sciences, Fudan University, Shanghai, China;

^2^State Key Laboratory of Genetic Engineering, School of Life Science, Fudan University, Shanghai, China;

^3^Department of Biochemistry and Molecular Biology, Mayo Clinic College of Medicine, Rochester, MN, USA;

^4^Laboratory of Cytobiology and Molecular Biology, The Affiliated Zhoushan Hospital of Wenzhou Medical University, Zhoushan, Zhejiang, China;

Correspondence: Dr. W Jiang, Key Laboratory of Metabolism and Molecular Medicine, The Ministry of Education, Department of Biochemistry and Molecular Biology, Shanghai Medical College, Fudan University, 130 Dongan Road, Xujiahui District, Shanghai 200032, China or Dr. L Yu, State Key Laboratory of Genetic Engineering, School of Life Science, Fudan University, 2005 Songhu Road, Yangpu District, Shanghai 200438, China.

E-mail: jiangw@fudan.edu.cn or longyu@fudan.edu.cn

^*^These authors contributed equally to this work.

**Supplementary materials**

**Supplementary figure legends**

Fig. S1 Predicted phosphorylation sites of NF90**.** Phosphorylation sites of NF90 were predicted and scored by the Group-based prediction system.

Fig. S2 Endogenous CDK2 interacted with NF90-Flag. **a** HEK293T cells were transfected with NF90-Flag plasmid. Cell lysates were collected for immunoprecipitation with M2 beads and subjected to WB. **b** The NF90 sequence contained the consensus CDK-phosphorylation motif (KSPXK). **c** NF90-Ser382 can be phosphorylated by CDK2/cyclin E1. The NF90 truncate (NF-D1) was purified and incubated with CDK2/cyclin E1 kinase in the reaction buffer. After incubation, the complex was run on a gel by electrophoresis and detected by mass spectrometry (MS). The phosphorylated peptide 372-PMEEDGEEKSPSK-386 with b- and y-ions indicated that S382 was a putative phosphorylation site. **d** Scheme of NF90 protein structure. Predicted CDK phosphorylation sites located in the nuclear localization signal motif.

Fig. S3 Deficiency of NF90-Ser382 phosphorylation prohibits cell proliferation. **a** Immunochemistry analysis of nuclear export of NF90-WT or NF90-S382A. GFP tagged NF90-WT or NF90-S382A were transfected into HeLa cells. After 24h, cells were fixed and stained with DAPI. **b** Detection of nuclear export of GFP-NF90 after transfection with CDK2/cyclin E1 or not. **c** Growth ability in Huh7 cells with the overexpression of vector, NF90-WT, and NF90-S382A. The cell proliferation assay was carried out over a 7-day culture period. Points, mean (n=6); bar, s.d. ** *p*<0.01; *** *p*<0.001. **d** HEK293T cells exogenously expressing wild type NF90 (WT), NF90-Ser382 phosphorylation deficiency mutant (S382A), and the control. Cyclin E1 protein expression was detected by WB. **e** The HEK293T cell proliferation assay was carried out over a 7-day culture period. Points, mean (n=6); bar, s.d. ** *P* < 0.01, *** *P* < 0.001.
