# Supplementary material for: NF90 stabilizes cyclin E1 mRNA through phosphorylation of NF90-Ser382 by CDK2
Source: Cell Death Discov. 2020 Jan 22;6:3. doi: 10.1038/s41420-020-0236-9 (PMC7026180; doi:10.1038/s41420-020-0236-9)
Supplement: Supplementary file 2 — Detailed Attribution of Authorship [file 41420_2020_236_MOESM2_ESM.pdf]

DECLARATION OF CONTRIBUTIONS TO ARTICLE

ADMC

Manuscript Number: CDDISCOVERY-19-0811-T

Journal Name: Cell Death Discovery (the 'Journal')

Proposed Title of the Contribution:

NF90 stabilizes cyclin E1 mRNA through phosphorylation of NF90-Ser382 by CDK2 (the 'Contribution)

Author(s):

Donglin Ding, Huixing Huang, Quanfu Li, Wenbo Yu, Chenji Wang, Haijie Ma, Jiaxue Wu, Yongjun Dang, Long Yu and Wei Jiang (the 'Authors')

For all covery articles, each person named as an author in the published version must be able to show he or she has contributed substantially to the article.

Authorship credit should be based on 1) substantial contributions to conception and design, acquisition of data, or analysis and interpretation of data; 2) drafting the article or revising it critically for important intellectual content; and 3) final approval of the version to be published. Authors should meet conditions 1, 2 and 3.

Any person who cannot be shown to have made a substantial contribution to the article cannot be listed as an author in the final version. The name of any person who is deemed to have made a minor contribution can, however, appear in the Acknowledgments section of the article.

Please complete the table below to indicate the contributions of all named authors to the manuscript.

Author Full Name:	Specification of Contribution to the Manuscript:
Donglin Ding	Performed experiments; cellected and analyzed data; wrote the manuscript
Huixing Huang	Performed experiments; cellected and analyzed data; wrote the manuscript
Quanfu Li	Performed experiments; cellected and analyzed data; wrote the manuscript
Wenbo Yu	Provided technical support; revised the manuscript.
Chenji Wang	Performed in vitro phosphorylation assay.
Haijie Ma	Performed IHC assay.
Jiaxue Wu	Drafted the aticle and revised the manuscript critically for important intellectual content
Yongjun Dang	Drafted the aticle and revised the manuscript critically for important intellectual content
Long Yu	Designed and oversaw the project, drafted the aticle.
Wei Jiang	Designed and oversaw the project; data analysis; drafted, worte and revised the manuscript

Please complete the table below to indicate the contributions of all named authors to the figures.

Figure 1:

Donglin Ding, Huixing Huang, Quanfu Li, Wenbo Yu, Chenji Wang, Jiaxue Wu, Yongjun Dang, Long Yu and Wei Jiang

Figure 2:

Donglin Ding, Huixing Huang, Quanfu Li, Jiaxue Wu, Yongjun Dang, Long Yu and Wei Jiang

Figure 3:

Donglin Ding, Huixing Huang, Quanfu Li, Jiaxue Wu, Yongjun Dang, Long Yu and Wei Jiang

Figure 4:

Donglin Ding, Huixing Huang, Quanfu Li, Wenbo Yu, Haijie Ma, Jiaxue Wu, Yongjun Dang, Long Yu and Wei Jiang

Figure 5:

Figure 6:

Signed for and on behalf of the Author(s):	Print Name:	Date:
Wei Jiang	Wei Jiang	11-18-2019
