# Supplementary figures and images for: NF90 stabilizes cyclin E1 mRNA through phosphorylation of NF90-Ser382 by CDK2
Source: Cell Death Discov. 2020 Jan 22;6:3. doi: 10.1038/s41420-020-0236-9 (PMC7026180; doi:10.1038/s41420-020-0236-9)

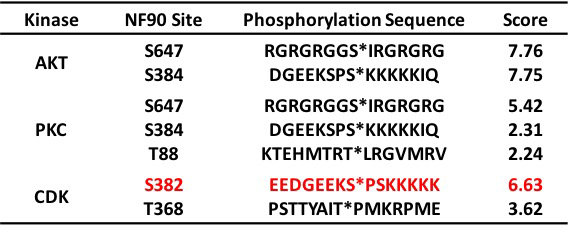

Supplement: Supplementary file 3 — Supplementary Material [file 41420_2020_236_MOESM3_ESM.tif]

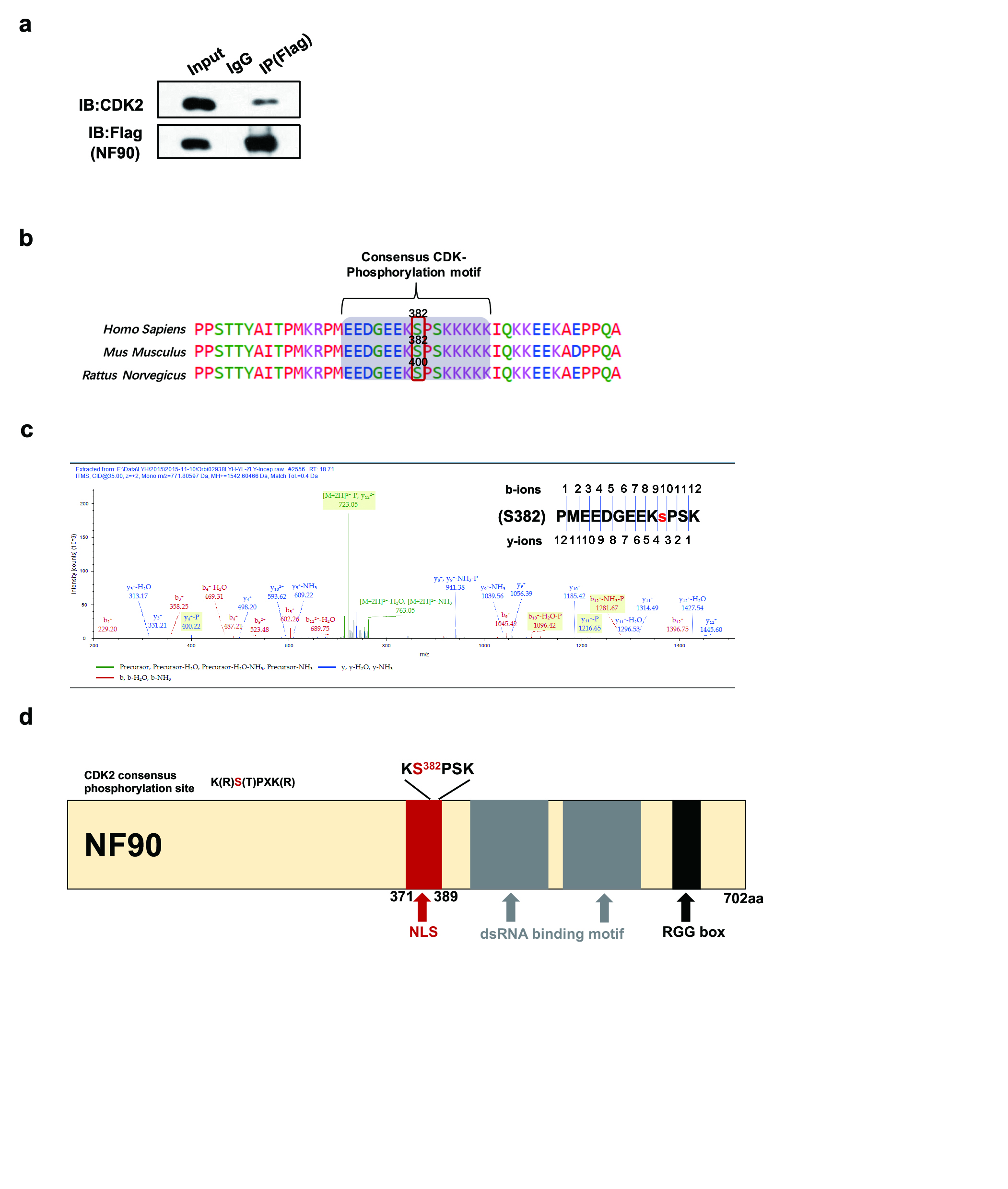

Supplement: Supplementary file 4 — Supplementary Material [file 41420_2020_236_MOESM4_ESM.tif]

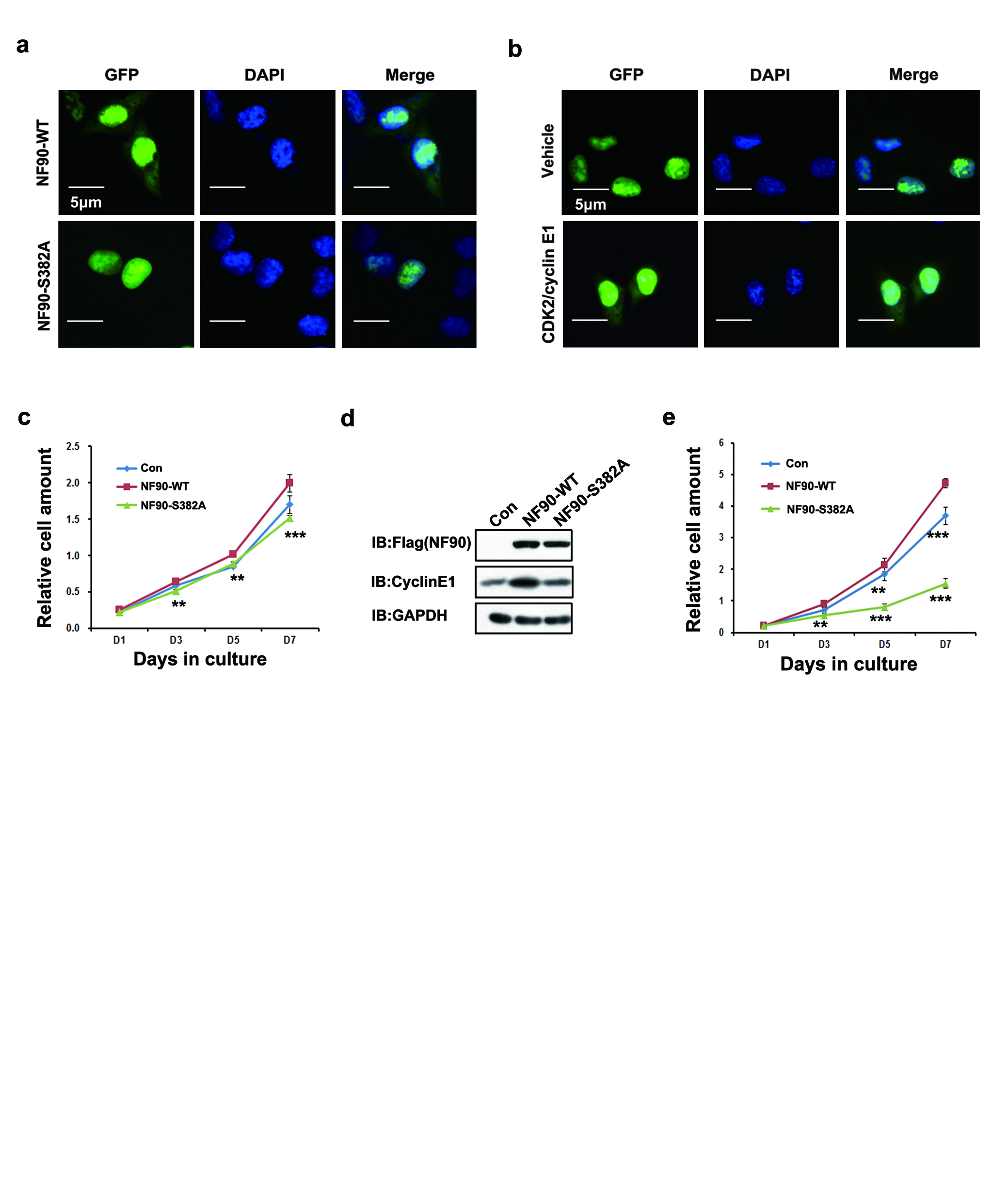

Supplement: Supplementary file 5 — Supplementary Material [file 41420_2020_236_MOESM5_ESM.tif]
